# Clinical Characteristics and Associated Factors of Mortality in Febrile Neutropenia Patients; a Cross Sectional Study

**Published:** 2019-07-27

**Authors:** Hamidreza Hatamabadi, Ali Arhami Dolatabadi, Ayda Akhavan, Saeed Safari

**Affiliations:** 1 Safety Promotion and Injury Prevention Research Center, Shahid Beheshti University of Medical Sciences, Tehran, Iran.; 2 Emergency Department, Imam Hossein Hospital, School of Medicine, Shahid Beheshti University of Medical Sciences, Tehran, Iran.; 3 Emergency Department, Shohadaye Tajrish Hospital, Shahid Beheshti University of Medical Sciences, Tehran, Iran.

**Keywords:** Chemotherapy-induced febrile neutropenia, infection, mortality, risk factors

## Abstract

**Introduction:**

The duration and severity of neutropenia directly correlate with the incidence of life-threatening infections. This study aimed to evaluate the clinical characteristics and associated factors of mortality in febrile neutropenia patients.

**Method:**

This retrospective cross sectional study was conducted on all febrile neutropenia patients who were admitted to oncology department of two educational hospitals, Tehran, Iran, from 2011 to 2016. Available patients’ data regarding baseline characteristics, treatment, and outcome were collected and analyzed using SPSS 21.

**Results:**

357 patients with the mean age of 50.9±17.7 years were studied (59.7% female). Mean white blood cell count of the studied patients was 715.1 ± 270.4 (100 – 1400) cells/mm^3^. The absolute neutrophil count (ANC) of all patients was <500 cells/mm3. The most frequent sources of malignancy in studied patients were gastrointestinal (35.9%), breast (22.4%), and sarcoma (15.7%), respectively. The mean time interval between initiation of treatment in ED and increase of ANC to > 500 cells/mm^3^ was 2.45 ± 2.1 (1 – 16) days. 186 (52.1%) subjects reached ANC>500 cells/ mm^3^ after 2-5 days of hospitalization. The rate of hospital mortality was 5.3% (338 (94.7%) survived). The correlation between gender (p = 0.11), temperature (p = 0.123), number of ED visits (p = 0.765), presenting clinical manifestation (p = 0.201), source of malignancy (p= 0.328), presence of metastasis (p = 0.69), positive urine culture (p = 0.45), positive blood culture (p = 0.62), time from last chemotherapy (p = 0.677), and time to reach ANC>500 cells/mm3 (p = 0.739) with mortality was not significant.

**Conclusion:**

Based on the findings of the present study, the rate of hospital mortality in patients with febrile neutropenia was 5.3%. Older age and lower white blood cell count were among the significant associated factors of mortality in this series.

## Introduction

The occurrence of fever in patients with cancer and reduced immune status is one of their subsequent problems. The lowest normal limit for circulating neutrophils is 1500 cells/mm3. The duration and severity of neutropenia directly correlate with the incidence of infections including those that are life-threatening. The incidence of infection finds a significant increase at the critical level of less than 500 cells/mm3 (1, 2). 

Neutropenia can be acquired or inherited. Its acquired type is common and is associated with different malignant conditions, radiotherapy and chemotherapy, and also idiosyncratic response to drugs such as phenothiazines, sulfonamides, penicillins, cephalosporins, and vancomycin (3). Neutropenia is the most prominent factor in the incidence of infection in patients undergoing chemotherapy or bone marrow transplantation, and fever is the most common symptom in these patients. About twenty years ago, gram-negative microorganisms were the most commonly isolated pathogens in patients with fever and neutropenia, but in recent years, gram-positive microorganisms have become more common and are found in about 60-50% of infections (4, 5). 

Today, fever in patients with a reduced level of immunity has become a major problem in hospitals. Due to the medical advances in the use of immunosuppressive drugs for the transplantation of various organs, the number of patients with fever and neutropenia has increased significantly (6). 

Those with persistent fever may be at risk for complications and need rapid medical treatment with antibiotics. Individuals with ANC > 500 cells/μl do not require treatment with Granulocyte - colony stimulating factor (G-CSF), but do require close monitoring for inflammation (7, 8). 

The mortality rate of febrile neutropenia has been reported from 5.4% to 15% in different studies (9-11). Patient characteristics, type of malignancy, age, race, prolonged neutropenia and infectious complications are stated as contributing factors of death in studies (12-14). 

We are faced with an increasing number of febrile neutropenia patients in our emergency departments and this study aimed to evaluate the clinical characteristics and associated factors of mortality in these patients.

## Methods

***Study design and setting***

This retrospective cross sectional study was conducted on all febrile neutropenia patients who were admitted to oncology departments of Imam Hossein and Shohadaye Tajrish Hospitals, Tehran, Iran, from 2011 to 2016. Available patients’ data regarding baseline characteristics, treatment, and outcome were collected and analyzed using SPSS 21. The study group adhered to the principles of medical ethics introduced by the Ministry of Health and the Declaration of Helsinki and legislation in the medical ethics committee of Shahid Beheshti University of Medical Sciences. In addition, the ethics committee of Shahid Beheshti University of Medical Sciences approved the protocol of the study (Ethics code: IR.SBMU.MSP.REC.1395.141). 

***Participants***

Data of all ≥ 15 years old febrile neutropenia patients who were admitted to the oncology departments of motioned hospitals were collected using census sampling method. Subjects with incomplete medical records were excluded.

***Data gathering***

**Data were recorded using a predesigned checklist containing demographic variables (gender, age), malignancy type, clinical presentation, time from last chemotherapy, type of received antibiotics, blood culture, urinary culture, cell blood count, neutrophil count, presenting temperature, number of ED visits, as well as outcome (survival, death). A trained third year emergency medicine resident was responsible for data gathering under direct supervision of an emergency medicine specialist.**

*Definitions*

**One time oral temperature of 38.3º C (101º F) or a persistent oral temperature > 38.0º C (100.4º F) for > 1 hour was defined as fever and ANC ≤ 500 cell/mm3 was defined as neutropenia in this study.**

***Statistical analysis***

Data were analyzed using SPSS version 21 and P<0.05 was considered significant. Findings were presented as mean ± standard deviation or frequency and percentage. T-test was used for comparing quantitative variables and chi-square or Fisher’s exact test for qualitative ones.

**Table 1:** Baseline characteristics of the studied patients

**Variables**	**Number (%)**
**Gender**	
Male	144 (40.3)
Female	213 (59.7)
**Age (Year)**	
< 20	27 (7.5)
21-40	80 (22.4)
41-60	126 (35.3)
> 61	124 (34.7)
**Source of malignancy**	
Lung	20 (5.6)
Gastrointestinal	128 (35.9)
Breast	80 (22.4)
Sarcoma	56 (15.7)
Genitourinary	35 (8.8)
Gynecology	17 (4.8.2)
Endocrine	13 (5.1)
Blood	8 (2.2)
**Clinical Manifestations**	
Dyspnea	27 (7.5)
Vomiting	58 (16.2)
Diarrhea	122 (34.2)
Cough	17 (4.8)
Weakness	54 (5.1)
Urinary symptoms	10 (2.9)
Abdominal pain	24 (6.7)
Others	23 (6.4)
**Time from last chemotherapy (day)**	
1-5	46 (12.9)
6-10	262 (73.4)
> 11	49 (13.7)
**Metastasis**	
Positive	93 (26)
Negative	264 (74)
**Number of ED visits**	
1	327 (91.7)
2	22 (6.1)
3	5 (1.4)
≥4	3 (0.8)

**Table 2:** Cultures and antibiotic therapy

**Variables**	**Number (%)**
**Antibiotics**	
Meropenem	201 (56.3)
Meropenem and Vancomycin	85 (23.8)
Imipenem	45 (12.6)
Imipenem and Vancomycin	5 (1.4)
Vancomycin	1 (0.2)
Tazocin	1 (0.2)
Ceftazidime	8 (2.2)
Others	11 (3.1)
**Blood Culture**	
Positive	43 (12.0)
Negative	314 (88.0)
**Urine Culture**	
Positive	18 (5.0)
Negative	339 (95.0)

**Table 3:** Comparison of different variables between survived and non-survived patients

**Variables**	**Survived**		**P value**
	**Yes (n = 338)**	**No (n = 19)**	
**Gender**			
Male	136 (97.7)	5 (2.3)	0.11
Female	208 (94.4)	8 (5.6)	
**Age (Year)**			
Mean ± SD	50.3 ± 17.6	62.1 ± 12.7	0.005
**Temperature**			
Mean ± SD	38.7 ± 0.4	38.5 ± 0.2	0.123
**Metastasis**			
Positive	89 (95.7)	4 (4.3)	0.69
Negative	255 (96.6)	9 (3.4)	
**Urine Culture**			
Positive	14 (100)	0 (0)	0.45
Negative	330 (96.2)	13 (3.8)	
**Blood Culture**			
Positive	38 (95)	2 (5)	0.62
Negative	306 (96.5)	11 (3.5)	
**WBC count (cell/mm3)**			
Mean ± SD	725.7 ± 268.7	526.3 ± 232.9	0.002
**Time to ANC>500 cell/mm3 (Days)**			
Mean ± SD	2.4 ± 2.1	2.7 ± 1.2	0.736
**Time from last chemotherapy (Days)**			
Mean ± SD	7.8 ± 2.7	8.1 ± 4.0	0.677

Data are presented as mean ± standard deviation (SD) or number (%). WBC: white blood cell; ANC: absolute neutrophil count.

## Results

***Baseline characteristics of febrile neutropenia patients***

357 patients with the mean age of 50.9±17.7 (16 – 88) years were studied (59.7% female). Mean temperature at the time of presentation to ED was 38.7 ± 0.4 (38.1 – 40) Celsius. Mean white blood cell count of the studied patients was 715.1 ± 270.4 (100 – 1400) cell/mm^3^. The ANC of all patients was <500 cell/mm3. Table 1 shows the baseline characteristics of the studied patients. The most frequent sources of malignancy in the studied patients were gastrointestinal (35.9%), breast (22.4%), and sarcoma (15.7%), respectively. 327 (91.7) cases had visited ED for the first time and 262 (73.4%) cases had presented 6-10 days from last chemotherapy. 43 (12.0%) cases with positive blood culture and 18 (5.0%) cases with positive urine culture were detected. 

***Outcome***

The mean time interval between initiation of treatment in ED and ANC increasing to > 500 cells/mm^3^ was 2.45 ± 2.1 (1 – 16) days. 186 (52.1%) subjects reached ANC>500 cells/ mm^3^ after 2-5 days of hospitalization. The rate of hospital mortality was 5.3% (338 (94.7%) survived). Table 3 compares different baseline characteristics of survived and non-survived subjects. The correlation between gender (p = 0.11), temperature (p = 0.123), number of ED visits (p = 0.765), presenting clinical manifestation (p = 0.201), source of malignancy (p= 0.328), presence of metastasis (p = 0.69), positive urine culture (p = 0.45), positive blood culture (p = 0.62), time from last chemotherapy (p = 0.677), and time to reach ANC>500 cell/mm3 (p = 0.739) with mortality was not significant. Mean white blood cell count of survived and non-survived patients was 725.7 ± 268.7 and 526.3 ± 232.9 cell/mm3, respectively (p = 0.002). The mean age of those who survived was significantly lower (table 3; p = 0.005).

## Discussion

**Based on the findings of the present study, the rate of hospital mortality in patients with febrile neutropenia was 5.3%. Older age and lower **white blood cell count were among the significant associated factors of mortality in this series. There was not any significant relationship between mortality and gender, temperature, number of ED visit, clinical manifestation, source of malignancy, presence of metastasis, positive urine culture, positive blood culture, time from last chemotherapy, or time to reach ANC>500 cell/mm^3^.

The mean age of patients in this study was 50.9 years and about 60% of cases were female. This finding is in line with Amini et al. study, which reported the mean age and gender distribution of neutropenia fever patients as 46 years and 52%, respectively (15) and also Fotokian et al. findings, which calculated the mentioned measures as 43 years and 50%, respectively (16). 

The most common sources of malignancy were gastrointestinal and breast, respectively; and diarrhea and vomiting were among the most frequent symptoms of patients in this series. In the study by Lakshmaiah et al. the most common sources of malignancy were lymphoma, leukemia, and germ cell tumors (17). In Hosseini et al. study, the majority of patients had lymphatic cancers (18). Hakim et al. reported blood-lymphatic cancers (16%) as the most common type of cancers, followed by solid tumors (19). Tamai et al. reported blood cancers and respiratory symptoms as the most common source of malignancy and patients’ complaint, respectively, in their series (20). These differences among the studies regarding the source of malignancy may be due to different distribution of malignancy types among countries and regions. 

We observed that almost 50% of individuals reached ANC>500 cells/mm3 after 2-5 days of treatment. Consistent with our finding, Cortés el al. revealed that the ANC of 56% of cases had improved and reached more than 500 cells/mm3 after four days (21). Another study showed that more than 80% of patients improve after a course of treatment (22). 

In this study, we observed that antibiotic type did not significantly influence patient outcome. Malik et al. found that use of the combination of ceftazidime and amikacin did not have any advantages over ceftriaxone monotherapy (23). These findings are also consistent with the results of the studies by Walsh et al., Ben et al., and Malik et al., because they concluded that single-agent regimens of ceftazidime and imipenem were equally effective in controlling neutropenic fever in patients with cancer (23-25). 

In our study, 11.2% and 3.9% of patients had positive blood and urine cultures, respectively. In many studies, bacteremia was reported in about 100% of patients (26, 27). In Hosseini's study, 13.7% of patients had a positive blood culture, and 13.7% had positive urine culture (18). In Soroush et al. study, the prevalence of positive blood culture and urine culture was 8% and 5%, respectively (28).

In a study on mortality of cancer patients with febrile neutropenia, Gray et al. concluded that adjusted risk of mortality increased 15% in cases experiencing febrile neutropenia (9). Chen et al., reported the death rate of neutropenia fever patients as 5.7% (10). Furthermore, Ahn et al. reported that 5.4% of patients admitted to hospital with neutropenia fever died (11).

Patient characteristics, type of malignancy, comorbidities, and infectious complications were among the most important associated factors of mortality in adult cancer patients with febrile neutropenia in one study (12). In the study by Swati et al. race, age, year of discharge, associated complications, and cancer type were significantly associated with higher mortality of these patients (13). In another study prolonged neutropenia, acute renal failure, nosocomial bacteremia, age >55 years, and monomicrobial bacteremia were among the significant predictive factors of mortality in hematologic patients with bacteremia (14). 

It is clear that the risk factors of mortality in febrile neutropenia are different among studies. This could be due to different economic, social, health, and case mix of these studies. It seems that each country or even each hospital should try to explore their own contributing factors of mortality and plan to reduce its rate via controlling the modifiable factors. 

**Limitation**

It should be noted that in the current study due to lack of access to patients, it was not possible to find other infectious sites.

## Conclusion:

Based on findings of the present study, the rate of hospital mortality in patients with febrile neutropenia was 5.3%. Older age and lower white blood cell count were among the significant associated factors of mortality in this series. 
